# Poor Association between Facial Expression and Mild Lameness in Thoroughbred Trot-Up Examinations

**DOI:** 10.3390/ani13111727

**Published:** 2023-05-23

**Authors:** Katrina A. Anderson, Ashleigh V. Morrice-West, Adelene S. M. Wong, Elizabeth A. Walmsley, Andrew D. Fisher, R. Chris Whitton, Peta L. Hitchens

**Affiliations:** 1 Equine Centre, Melbourne Veterinary School, Faculty of Science, University of Melbourne, Werribee, VIC 3030, Australia; 2 Avenel Equine Hospital, 34 Ferguson Lane, Avenel, VIC 3664, Australia; 3 Animal Welfare Science Centre, Faculty of Science, University of Melbourne, Parkville, VIC 3052, Australia

**Keywords:** equine, lame, musculoskeletal injury, orthopaedic pain, pain score, horse grimace scale, facial expression

## Abstract

**Simple Summary:**

Fatal injuries in Thoroughbred racehorses typically occur due to an accumulation of bone damage, however, detecting their impending onset can be difficult as there are often no overt signs. In other horse populations, facial grimacing has been shown to be associated with orthopaedic pain. This study, therefore, aimed to investigate facial expressions of Thoroughbred racehorses at the trot to identify if there were subtle signs of pain in mildly lame compared to non-lame horses. Two independent observers assessed 22 facial expression parameters using still photographs of the head from video-recorded trot-ups. There were few expressions associated with lameness except for more exposed whites of the eye in lame horses. Thus, facial pain scoring may not be adequate for the detection of subtle lameness in racehorses who work in a racing stable environment.

**Abstract:**

Musculoskeletal injuries in racehorses are difficult to detect prior to catastrophic breakdown. Lameness is commonly attributed to orthopaedic pain in horses, therefore, subtle lameness may be a pre-clinical sign of injury and, if identified early, could allow for preventative intervention. Our objective was to determine if facial expressions could be used to detect mild lameness as an indicator of orthopaedic pain in ‘fit to race’ horses. The Horse Grimace Scale (HGS) and the facial expressions in ridden horses (FEReq), were used to score images (n = 380) of mildly lame (n = 21) and non-lame (n = 17) Thoroughbred horses by two independent observers. Using an Equinosis Lameness Locator^®^, the lameness status of each horse was determined according to published thresholds [forelimb lameness (>|14.5 mm|) and hindlimb lameness (>|7.5 mm|)]. Inter and intraobserver reliability were assessed using two-way random-effects models. Univariable associations between lameness and facial expression parameters were identified using logistic and linear regression. Interobserver reliability was moderate (κ 0.45; 95% CI 0.36, 0.55). Horses with moderate mouth strain (HGS) and tense and extended upper lip (FEReq) were less likely to be lame (*p* = 0.042 and *p* = 0.027, respectively). Exposed sclera was associated with lameness (*p* = 0.045). Higher orbital tightening (HGS) scores were associated with a lower degree of maximum head amplitude (HDmax) lameness (*p* = 0.044). Tension and moderate tension above the eye, for the HGS and FEReq scores, were associated with increasing amplitude of HDmax (*p* = 0.048 and *p* = 0.034, respectively). Inconsistent associations between lameness status and HGS and FEReq scores may limit the potential use of the facial expression for the prediction of mild orthopaedic pain during pre-race lameness examinations. More objective parameters associated with mild orthopaedic pain should be explored.

## 1. Introduction

Equine lameness is defined as an alteration of normal gait due to a functional or structural disorder of the locomotor system and is commonly attributed to orthopaedic pain [1,2]. While lameness remains the most commonly reported reason for failure to train and reduced racing performance [3,4], numerous studies have revealed the high incidence of horses with gait asymmetries not detected by owners or trainers [5,6,7].

Many orthopaedic injuries in racehorses are the result of accumulated bone microdamage over time [8], thus there is a spectrum of injury ranging from subclinical (i.e., no appreciable signs) to severe lameness (i.e., non-weight bearing). Whilst overt signs of lameness indicative of pathology can be readily identified and, therefore, promptly addressed, subtle signs potentially associated with the onset of underlying injury are difficult to detect. To minimise the likelihood of the progression of bone injury to overt fracture, more sensitive methods for evaluating the accumulation of bone fatigue, especially during its early stages, are required. When microdamage accumulates faster than it can be repaired, the integrity of the bone will eventually be overcome, resulting in gross injury such as fracture [9]. A sudden onset of moderate to severe lameness that improves quickly is a key indicator of a stress fracture in racehorses [10]. But, since horses that fracture do not typically demonstrate clinical signs of an impending problem, the accurate detection of horses at risk is challenging.

Lameness is most commonly assessed using subjective visual evaluation of (a)symmetry between the left and right side during the gait in-hand at the trot [11]. However, agreement in scores between scales including the American Association of Equine Practitioners (AAEP) lameness scale, numerical rating and verbal rating, is moderate to low [12,13]. Objective tools can be used to evaluate gait symmetry and improve reliability [12]. For example, kinetic gait analysis has shown a potential to detect reduced force prior to subjectively observed lameness [14]. Additionally, wireless inertial sensor units for evaluation of lameness on all limbs over the ground have proved repeatable for use in a clinical setting [15]. However, for everyday use such as in stabling environments, efficient routine methods of detection are required.

Studies have reported up to 75% of horses considered to be non-lame (“sound”) fell outside original inertial measurement unit thresholds of asymmetry [7,16]. This suggests that asymmetric horses can compete successfully, and that trainers and riders may consider horses sound in the presence of mild lameness [6,17]. Objective measurements previously categorised a horse as lame when left and right height differences were greater than thresholds >6 mm for head and >3 mm for pelvis [18]. However, thresholds developed by Pfau et al. were determined to better encompass horses ‘fit to race’, with the acceptable differences in head height between the left and right sides increased from <6 mm to <14.5 mm and pelvic displacement from <3 mm to <7.5 mm [19,20]. Recent discussions have highlighted the need to better understand whether asymmetry is due to a pathological condition and manifestation of pain [21,22]. Following the detection of gait asymmetry, the interpretation of whether it represents orthopaedic pain requires further work up and can be confirmed using diagnostic analgesia [21,23,24].

Scales grading horses’ facial expressions have been developed to objectively evaluate pain from various sources [25,26,27]. For example, the Horse Grimace Scale (HGS) was validated to quantify pain in horses’ post-castration surgery [26,28] and to assess horses after orthopaedic surgery [29]. Facial expressions in ridden horses (FEReq) have also been demonstrated to differentiate lame and non-lame horses [27,30]. When assessed using the Ridden Horse Pain Ethogram (RHpE), an adaptation of FER(eq) [31], ridden horses that were lame displayed certain behavioural markers more often than sound horses, and two markers (sclera exposed, bucking or kicking backward) were exclusively observed in lame horses [23]. However, in a study of eight non-Thoroughbred horses with induced lameness, a combination of postural behaviours and facial expressions in horses at rest was necessary to predict movement asymmetry scores, suggesting that facial expression alone might not be sensitive enough to detect mild orthopaedic pain [32,33].

The objective of this study was to assess the validity and clinical applicability of the HGS and FEReq during a standard in-hand trot-up examination in order to assess if facial expressions could be useful in differentiating between mildly lame and non-lame Thoroughbred horses where overt signs of lameness may not be present. To our knowledge, this is the first study to assess the relationship between facial expressions and orthopaedic pain in actively racing Thoroughbred horses. We hypothesised that high facial pain scores would be associated with lameness above that considered appropriate for racing.

## 2. Materials and Methods

A total of 38 Thoroughbred horses were recruited: 12 horses from the University of Melbourne Equine Centre admitted for lameness and poor performance, and 26 horses, deemed fit for racing from the training yard of one Victorian registered trainer. Information for each horse including age, sex and racing history was obtained from the official repository for racing information in Australia (Racing Australia; https://www.racingaustralia.horse/; accessed on 28 August 2020).

### 2.1. Lameness Examination

Objective lameness assessments were performed using a body-mounted inertial sensor system (Lameness Locator^®^, Equinosis LLC, St. Louis, MO, USA). Each horse was instrumented with two single-axis acceleration sensors and one single-axis gyroscope sensor (3.8 × 2.5 × 1.3 cm^3^; 30 g). Sensors were turned on with a magnetic switch prior to attachment. One acceleration sensor was placed on the poll using a head bumper attached to the halter. The second accelerometer was placed over the tuber sacrale attached with 3M dual lock tape and secured with an additional ~20 cm strip of 5 cm width Leukoplast Waterproof Tape covering the sensor and secured laterally onto the skin. The gyroscope sensor was placed on the dorsal surface of the right forelimb pastern using the manufacturer’s pouch and strap. Total instrumentation time was <2 min. Data obtained by the three sensors were wirelessly transmitted in real time from the Lameness Locator (8 bits at 200 Hz) to a tablet, and a series of motion analysis algorithms previously developed were automatically performed to evaluate gait and provide diagnosis [34,35,36]. Lameness reports were developed using asymmetry recorded by the Lameness Locator for the following variables: maximum head displacement (difference between maximum heights of the head after stance of forelimbs); minimum head displacement (difference between minimum heights of head during stance of forelimbs); total difference head; maximum pelvis displacement (difference between maximum heights of the pelvis after stance of hindlimbs); minimum pelvis displacement (difference between minimum heights of pelvis during stance of hindlimb) [15]. Movement symmetry was determined by calculating differences in minimum and maximum values for both head and pelvic displacement during a full stride [15,35,37]. Horses were considered lame if thresholds were >14.5 mm for head and >7.5 mm for pelvis displacement, under which horses were categorised as ‘fit to race’ [20].

All horses underwent a lameness examination which included an. in-hand trot-up of over twenty meters (>25 strides) on a hard surface in a straight line. All horses wore a bit and bridle during the trot-up. Lameness was evaluated subjectively by one of four veterinarians at the time of data collection based on the AAEP lameness scale (0–5); where zero is no lameness observed under any circumstances and five is non-weightbearing lame. Horses were additionally video recorded using one high-definition camera (Panasonic HC-V770M) set up at a position lateral to the trotting area (at the centre of the twenty meters) and panning to capture the horse from lateral, craniolateral and dorsolateral views for the duration of the trot-up. The video was used only for the purposes of pain score assessment and not for assessment of lameness. Trot-ups were video recorded at the time of lameness examination for hospital-admitted equine patients and at the time of routine weekly trot-up for the horses in training. To randomise selection, every third horse in the training stable was recruited. One horse was manually re-classified as sound, based on the AAEP lameness scale, despite forelimb thresholds exceeding 14.5 mm, because for the duration of trot-up, the horse displayed excessive behavioural head movements (head tossing).

### 2.2. Image Processing

Using the video recording of the gait examination, still shots were taken at midstance—the point of maximum force on the horse’s limbs—for every footfall between a marked area (5–15 m) using the event logging software, BORIS [38,39]. A random number generator was used to allocate a unique identification number to each horse and image. Each image was documented as the stance of the left forelimb or right forelimb. Images were cropped so that only the head and part of the neck were visible to observers. This was to prevent the observers from being influenced or biased by other indicators of pain, the position of the body and/or the background location [26,27,30,40]. Images of poor quality (pixilated, dark), where the entire head was not visible or where obvious tension on the lead was present, were excluded from the collection. Then for each horse, ten images were randomly selected and used to create a slideshow with one image per slide (n = 380 images; Microsoft PowerPoint) [41]. Slides were shuffled so there was no order to images to avoid the observer’s scores being influenced by consecutive images of the same horse.

### 2.3. Pain Assessment

Two separate subjective pain scoring systems were employed: (1) Horse Grimace Scale (HGS) [26] and (2) ethogram using facial expression in ridden horses (FEReq) [27,30]. Two independent observers, blinded to horse and lameness categorisation, conducted evaluations twice, 40 to 50 days apart. Both observers had training in animal welfare and behaviour, but with limited horse experience. A training manual, adapted from previously developed scales [26,27,30,40], was provided on how to score pain from facial expression parameters (Supplementary Item S1). The instructions included an emphasis on the importance of recording the presence of each facial expression individually without any regard for the presence of other pain-related parameters. For images where observers could not see or grade a parameter, rather than speculating, observers were instructed to score as “cannot tell”.

The HGS includes six facial action units (FAUs): stiffly backwards ears; orbital tightening; tension above the eye area; prominent strained chewing muscles; mouth strained and pronounced chin; strained nostrils and flattening of the profile. For each image the observer scored each individual FAU using a 4-point scale (0 = not present, 1 = moderately present, 2 = obviously present or CT = cannot tell) [26]. Additionally, the observer was asked to make an overall pain judgement using a simple descriptive scale (SDS; no pain, mild, moderate and severe) demonstrated to be highly repeatable [26,42,43].

We adapted the FEReq ethogram to exclude two parameters (position of head and bit) as could not account for influence from tension on the bit from the handler [27,30]. Observers were instructed to confirm the presence or absence of each facial expression parameter. A pain score, blinded to observers, was allocated to each parameter, previously determined to correlate to severity of pain Appendix A (Table A1) [30].

### 2.4. Statistical Analysis

The overall proportion agreement and intraobserver and interobserver reliability were calculated to determine agreement within each observer and between observers on the same images, respectively, and reported as intraclass correlation coefficients in an unbalanced two-way random-effects design [44]. Values were classified as <0.20 poor agreement, 0.21 to 0.40 fair, 0.41 to 0.60 moderate, 0.61 to 0.80 good and >0.80 very good [45]. Observer 1 scored 36 horses on the first evaluation (images n = 360), observer 2 scored 38 horses on the first evaluation and both observers scored all 38 horses on the second evaluation (images n = 380).

Univariable logistic regression models for the binary outcome lameness (lame = 1; non-lame = 0) and linear regression models for the continuous outcome asymmetry values were performed to investigate associations with facial expression parameters, adjusting for clustering on horse to account for multiple observations of each horse. The absolute values of minimum and maximum amplitude for head and pelvis were used as a continuous outcome for degree of lameness. Missing values were excluded when calculating the sum of HGS and FEReq scores. A pairwise correlation coefficient was generated to determine the relationship between total HGS and total FEReq scores. Statistical significance was set at *p* < 0.05. Statistical analysis was performed using Stata 15 (StataCorp. 2017. Stata Statistical Software: Release 15. College Station, TX, USA: StataCorp LP).

## 3. Results

The horses ranged in age from two to nine years (mean: 3.7 years, sd: 1.5 years) and comprised 13 females, 13 gelded males and 12 entire males. Horses were allocated as ‘fit to race’ (n = 22) or lame (n = 16; Table 1) using the Equinosis Lameness Locator and proposed thresholds [20]. Using original lameness thresholds [18], 7.9% of horses (3/38) were considered sound. Where the horse was trotted up in-hand over twenty meters on a hard surface in a straight line, on the AAEP scale veterinarians reported n = 4 (10.5%) horses were grade 0, n = 17 (44.7%) were grade 1 and n = 17 (44.7%) were grade 2. There were no horses graded AAEP > 2. Lameness did not differ by sex, with 31.3% (5/16) of lame horses being female, 43.8% (7/16) of lame horses being geldings and the remaining 25.0% (4/16) of lame horses being entire males. Lameness did not differ by age, number of race starts or prizemoney.

Intraobserver reliability was higher than interobserver reliability for all facial regions. Intraobserver reliability was very good for HGS scoring of ear position (κ 0.83) and orbital tightening (κ 0.80), and for FEReq scoring of the tongue (κ 0.84), mouth (κ 0.83) and stiffly backward ears (κ 0.83). For all remaining behaviours, agreement within observers was moderate to good (Table 2).

Using the HGS, interobserver reliability was good for orbital tightening (κ 0.76), but poor for strained nostrils (κ 0.15). Interobserver reliability using the FEReq was good for eye tension (κ 0.76), tongue (κ 0.75) and sclera (κ 0.72) and poor to fair for eye shape, stare, tension above the eye, nostrils tension, a wrinkle between nostrils, wrinkles ventral to nostrils, upper lip and nose twisted. All behaviours with good to very good intraobserver reliability had moderate to good interobserver reliability.

Observers were unable to score wrinkles between nostrils in 38.1% of images (572/1500), followed by prominent chewing muscles (28.1%; 422/1500), lower lip (15.1%; 226/1500), mouth strain (12.3%; 184/1500) and nostril tension (10.2%; 153/1500).

Associations between facial expression parameters and lameness are presented in Table 3 (HGS) and Table 4 (FEReq). One association was identified using the HGS; horses with moderately present mouth strain were less likely to be lame (OR 0.51; 95% Cl 0.27, 0.97; *p* = 0.042). There were two associations using the FEReq; horses with exposed sclera were more likely to be lame (OR 3.33; 95% CI 1.03, 10.79; *p* = 0.045), and horses with tense and extended upper lip were less likely to be lame compared to those with a relaxed lip (OR 0.40; 95% Cl 0.18, 0.90; *p* = 0.027). There were no associations observed using either the HGS or FEReq grading systems between ear position and lameness (*p* > 0.05).

Associations between individual facial expression parameters and the degree of lameness on a continuous scale are presented in Supplementary Table S1. Moderate orbital tightening (HGS) and eye tension (FEReq) were associated with lower HDmax values, albeit eye tension did not reach significance (*p* = 0.044 and *p* = 0.054, respectively). Tension or moderate tension above the eye, for both the HGS and FEReq scores, was associated with greater HDmax values (*p* = 0.048 and *p* = 0.034, respectively). The presence of wrinkles ventral to nostrils was associated with lower HDmin values (*p* = 0.034). Prominent chewing muscles were associated with lower HDmin values (*p* = 0.027), but greater PDmax values (0.014). Visibility of the tongue was associated with lower degree of forelimb lameness (HDmin and HDmax) and greater degree of hindlimb lameness (PDmin and PDmax) (*p* < 0.01), although the majority of observations were scored as tongue in, not seen (1273/1411; 90.2% observations).

There was no association between the total HGS score and lameness (lame n = 381 and ‘fit to race’ n = 508; median 3 and 4; mean ± s.d. 3.42 ± 1.82 and 3.86 ± 2.02, respectively; *p* = 0.235). There was no association between the total FEReq score and lameness (lame n = 235 and ‘fit to race’ n = 320; median 7 and 7; mean ± s.d. 7.10 ± 3.49 and 6.94 ± 3.58, respectively; *p* = 0.235). Total HGS and total FEReq scores were significantly correlated (rho 0.766; *p* < 0.001).

## 4. Discussion

Out of 22 facial expression parameters evaluated, only three were associated with lameness in Thoroughbreds at a level that was considered unfit to race, and two of the three associations were in the opposite direction to that hypothesised. When visible, moderate mouth strain and a tense or extended upper lip were observed more commonly in images of ‘fit to race’ horses, whereas exposed sclera was more often observed in images of lame horses. The total facial scores in HGS and FEReq systems were not different between ‘fit to race’ and lame horses. Although consistent within each observer, 45% (10/22) of the parameters lacked repeatability between observers (interobserver reliability < 0.4).

That there were differences in the classification of horses that were initially selected based on whether they were in race training (n = 26) or presented to a veterinary hospital (n = 12), and subsequently reclassified as ‘fit to race’ (n = 22) or lame (n = 16) based on Equinosis Lameness Locator thresholds [19,20] highlights the difficulties in identifying subtle pain and mild lameness.

In contrast with the findings of the current study, Dyson, Berger, Ellis and Mullard [30] determined total facial scores were significantly higher for lame-ridden horses (mean, 8.7 ± 0.15) compared to sound-ridden horses (mean 6.1 ± 0.32) [30]. However, during development of the FEReq, weightings were allocated to regional facial expressions based on greater prominence in lame horses; the eyes, ears and nostrils were more heavily weighted than those of the lips, muzzle or tongue-in-ridden horses. Further, that study included additional parameters related to riding not considered here. The exposed sclera was considered a strong indicator of lameness by Dyson, Berger, Ellis and Mullard [23], supported by the findings of this current study.

‘Fit to race’ horses did not have lower overall facial pain scores compared with horses considered lame or unfit to race. Thus, assessment of facial expressions during a trot-up examination for Thoroughbred racehorses in a training stable environment was not adequate for detecting mild lameness. Lameness severity for horses recruited for this study was mild, with no horse scoring greater than two on the AAEP lameness scale. However, horses in this present study were not subjected to all conditions recommended under the AAEP scale (e.g., weight-carrying, circling, inclines and different surfaces). The ability for FEReq to determine the presence of musculoskeletal pain was validated previously in lame-ridden horses [30], and although lameness scores were not stated, it is unlikely they would have been graded more than two given they were ridden. However, it is possible that facial expression in lame horses is more overt when they are ridden. In another study, postural behaviours at rest were found to be most strongly associated with movement asymmetry in horses with induced mild orthopaedic pain but facial expressions less so. However, HGS parameters positively associated with pain did include orbital tightening, stiffly backward ears, tension around the eye area and nostrils [32]. Particularly for mild lameness, a combination of behaviours in horses at rest, such as posture, head position, location in the stable, focus, interactive behaviour and facial expressions, may improve the detection of orthopaedic pain [32]. A recent study has demonstrated the ability to detect mild to severe lameness using the Ridden Horse Pain Ethogram but also found that lameness was more apparent when ridden compared to movement in-hand, as well as at a canter compared to at a trot [46]. Therefore, it could be the case that milder pain associated with mild lameness may only be evident during ridden exercise but less detectable during an in-hand trot-up exercise as was the case in our study.

Contrary to our hypothesis, we observed evidence of strain and tension around the mouth and lips in horses categorised as ‘fit to race’. A previous study found no significant differences for lips and muzzle parameters between lame and sound ridden horses [30]. This facial region may be insensitive or nonspecific as a predictor of orthopaedic pain or may occur only at very low levels of discomfort. Horses experiencing discomfort from a range of sources may also hold their ears backward and lowered [26,27,40], but we did not observe ear position associations in the current study.

An alternate explanation for our findings is that some facial expressions occur due to other factors such as environmental or handler interactions during the trot-up rather than an expression of discomfort. Facial expressions are not specific for orthopaedic pain [26] and may be confounded by other sources such as generalised stress or gastric ulceration, particularly in Thoroughbred racehorses [47], the investigation of which was outside the scope of this study. Previous studies validated the HGS with values shown to increase following surgical intervention [26] and by demonstrating a reduction in pain score in horses following analgesia with acute laminitis [43]. A baseline level of total HGS (~2 out of a maximum score of 12) in a presumed healthy population of horses [26], likely accounts for environmental influences on the presence of the measured facial expressions. The total HGS score for both groups in the current study (median 3–4) was only slightly elevated from this baseline value. Given the high number of parameters scored ‘cannot tell’ in our image dataset (n = 1007), it is likely that such a large proportion of missing data would result in the total HGS scores underestimating the horse’s true HGS. Because of this, total score findings should be interpreted with caution, with a focus on individual parameters instead. Irrespective, our study demonstrates that the evaluation of facial expressions may not be suited for differentiating between mildly lame and non-lame horses.

The interobserver reliability of facial parameters for this study was lower compared to previous research [26,30,43]. Of HGS parameters, our study had the lowest agreement for strained nostrils (0.15), reported to be moderate (0.58) in previous validation studies and the lowest of all HGS parameters [26]. The FEReq was previously validated to recognise musculoskeletal pain by only one observer and, therefore, we are unable to compare the interobserver reliability of our findings [30]. The two observers in our study had experience with animal behaviour and welfare but were not specific to horses. It is possible this could contribute to different interpretation of facial expressions. Additionally, we cannot rule out that a limited range of scores, as would be expected in a cohort of only mildly lame horses, may have contributed to reduced reliability. A previous study showed no differences in the scoring of observers regardless of professional background [27]. However, in another study, observers with no horse experience that received 30 min of standardised training did not reach good agreement with an HGS expert for most facial expressions, with the exception of stiffly backward ears and orbital tightening [48]. Intraobserver reliability was higher than interobserver reliability for every facial expression scored, ranging from fair to excellent (0.49 to 0.84), suggesting that although reliability was in general only fair to moderate between observers, the ability for one observer to repeat scores was generally high. Therefore, there is potential for one observer to monitor and identify changes in horses’ expressions over a period of time. A change in expression, particularly of factors associated with lameness such as exposed sclera, or an increase in an individual’s total score over time, may be more useful in identifying horses that are developing orthopaedic pain than a single assessment. Identification of more subtle signs of pain may be improved by using more experienced HGS and FEReq observers, improved image quality and/or more objective measurements of facial expression. Alternatively monitoring facial scores remotely, for example utilising video cameras when horses are stabled may be more sensitive for detection of pain, as recent studies have demonstrated horses do not as readily display signs of discomfort when they know they are being observed [49]. Temporal information has previously been found to be important for interpreting facial expressions of pain in horses [40]. Exploration of automatic facial expression identification or machine learning models trained to identify features in horses known to be in pain may also remove the subjectivity of individual observers. This method has been piloted successfully using EquiFACS (Facial Action Coding System) data, with a neural network outperforming blinded veterinarians in experimentally induced temporal pain assessment of horses using videos [50].

A large percentage of observations were recorded as ‘cannot tell’, there was a substantial difference in observations not scored between observers (n = 2805 versus 271 not scored), and observers had the most difficulty scoring facial parameters located in the muzzle region. Horses in our study were recorded from a lateral aspect of the trotting track, therefore, images were predominantly a lateral view of the horse, potentially explaining the high percentage of wrinkles between nostrils not scored. In ridden horses, wrinkles between nostrils were recorded as ‘cannot tell’ in 70% of images [27]. Randomly selected images included different angles and some craniolateral images as the horse approached the mid-point closest to the camera. Prominent chewing muscles scored as ‘cannot tell’ in 28% (422/1500) of images may be explained by these different angles or the placement of the bridle obscuring this region. In addition to locomotion, these horses were wearing bridles and head pieces (with accelerometer attachment) potentially interfering with the observer’s ability to score. Multiple cameras and alternate views may provide clearer images of features that may not be appreciated by a lateral or craniolateral view.

There were several limitations to this study. Based on findings from previous studies [30], we had power of >80% to detect a difference in scores from our two groups with ten static facial images each; however, given we observed no discernible differences between our mildly lame and ‘fit to race’ horses, significant refinement of the methodology and a much larger sample size would be required to detect minute differences in facial expression scores. Due to the large number of statistical tests performed it is possible that significant findings could be due to chance. Not all trot-ups recorded more than 25 strides (mean 30; range 12–65) as recommended [18]. This was due to technical difficulties or horses not cooperating, typical challenges of performing evaluations in a busy training facility. However, previous research has suggested that stride-by-stride variation was not affected by the number of strides collected [15]. It was difficult to maintain a level of consistency between trot-ups due to varying weather, handlers and the temperament of horses. A horse that throws its head around repeatedly during a trot-up, due to demeanour, environmental stimuli or in response to a handler will result in a wide variation in values for head displacement, although the Equinosis Lameness Locator identifies and removes outlier movements. Accelerometers are repeatable for the measurement of asymmetry of pelvic (≥0.93) and head displacement (≥0.89) [18]. However, a longitudinal study demonstrated racehorses had median weekly differences of 4 to 8mm, indicating potential limitations of using cross-sectional data based on inertial measurement unit gait analysis to classify horses as ‘fit to race’ [19]. Additionally, cases of symmetric bilateral lameness may be incorrectly evaluated as sound by the Equinosis Lameness Locator [51]. While previous studies have demonstrated that lameness was more apparent ridden as opposed to in-hand [52], the purpose of this study was to evaluate the usefulness of the facial pain scored as a tool to augment pre-race trot examinations where lameness might not be overtly evident. Lastly, variation in pain scores in individual horses may have been affected by the timing of the photo. The nature of sampling at one timepoint may lead to misinterpretation of behaviour overall. Some horses appeared to have their eyes closed which may be interpreted as orbital tightening; however, the images may have captured the horse whilst blinking. Video recordings are sensitive to the identification of reduced levels of pain following diagnostic analgesia [24], and, therefore, may be more clinically applicable to scoring orthopaedic pain in horses during trot-up examinations. Observers in this study scored facial parameters based on their presence or absence irrespective of individual horse personality or behavioural characteristics and without knowledge of any asymmetrical face or bone structure variations. Objective measurement of the individual’s baseline behaviour may help better define changes in behaviour or abnormal expression.

## 5. Conclusions

Few differences in facial pain parameters were observed between lame and ‘fit to race’ horses. Contrary to our hypothesis, facial expressions generally did not differentiate between Thoroughbred racehorses considered ‘fit to race’ from those that demonstrated mild lameness during in-hand trot-ups, therefore, limiting the usefulness of this technique as a potential tool for pre-race examination. Longitudinal investigations to monitor changes in racehorse lameness over time may be required to elucidate potential relationships with facial expressions or other behavioural or demeanour changes. This may be a more useful indication of impending orthopaedic injury. There were inconsistencies in both the classification of lameness by veterinarians and the inertial sensors and for facial expressions by trained animal welfare specialists, which highlights the need for the use of more objective assessment.

## Figures and Tables

**Table 1:** Number or mean (standard deviation) of lame and ‘fit to race’ horses from total sample of Thoroughbred horses according to the Equinosis Lameness Locator measurements in trot-up examinations (N = 38).

	Fit to Race	Lame	*p*-Value
n	22	16	
Age	3.4 (1.0)	4.1 (1.8)	0.282
Race starts	4.3 (6.2)	2.3 (3.3)	0.203
Prizemoney	$111,553 (135,827)	$170,375 (414,096)	0.233
Sex			0.606
Female	8	5	
Gelding	6	7	
Entire	8	4	
AAEP ^†^ threshold	21	17	0.976
AAEP ^†^ score			
0	3	1	0.566
1	11	6	
2	8	9	

^†^ American Association of Equine Practitioners.

**Table 2:** The inter and intrareliability (κ) of two independent observers scoring facial expression parameters from lateral images of Thoroughbred horses’ heads (n = 38) at two time points (40 to 50 days apart). A total of n = 1500 assessments of 380 images were conducted using two grading systems; the Horse Grimace Scale (HGS) and the facial expressions in ridden horses (FEReq).

	Behaviour Parameter	Agreement % ^†^	Observations Not Scored ^‡^		Interobserver Reliability	Intraobserver Reliability
			Observer 1	Observer 2		
	Stiffly ears	61.8	0	19	0.57 (0.41, 0.68)	0.83 (0.79, 0.87)
	Orbital tightening	83.6	0	101	0.76 (0.73, 0.80)	0.80 (0.76, 0.83)
	Tension above the eye	54.6	8	111	0.48 (0.44, 0.55)	0.60 (0.53, 0.64)
**HGS**	Prominent chewing muscles	71.6	2	420	0.45 (0.47, 0.59)	0.55 (0.22, 0.44)
	Strained nostrils	50.9	5	138	0.15 (0.03, 0.28)	0.63 (0.34, 0.86)
	Mouth strained	62.7	18	166	0.60 (0.56, 0.66)	0.63 (0.54, 0.66)
	Overall pain	46.2	0	20	0.25 (0.18, 0.33)	0.45 (0.34, 0.56)
	Ears (position)	52.2	0	23	0.69 (0.64, 0.73)	0.83 (0.80, 0.85)
	Stare (normal or intense)	64.5	8	141	0.28 (0.19, 0.37)	0.47 (0.29, 0.59)
	Eye tension	83.6	0	109	0.76 (0.73, 0.80)	0.80 (0.75, 0.82)
	Sclera (exposed or not exposed)	97.4	1	14	0.72 (0.68, 0.76)	0.78 (0.74, 0.81)
	Eye shape (almond or round)	57.4	0	119	0.23 (0.09, 0.37)	0.68 (0.48, 0.84)
	Tension caudal to eye	76.8	7	93	0.54 (0.49, 0.60)	0.66 (0.60, 0.69)
	Tension above eye	70.2	10	120	0.39 (0.32, 0.50)	0.70 (0.62, 0.75)
**FEReq**	Nostril tension	57.0	5	148	0.16 (0.06, 0.29)	0.56 (0.26, 0.79)
	Wrinkle between nostrils	67.0	163	409	0.14 (0.12, 0.35)	0.73 (0.37, 0.80)
	Wrinkles ventral to nostrils	84.4	9	91	0.34 (0.28, 0.43)	0.59 (0.50, 0.64)
	Upper lip	64.7	6	128	0.38 (0.33, 0.47)	0.49 (0.41, 0.53)
	Lower lip	77.1	23	203	0.54 (0.47, 0.61)	0.62 (0.50, 0.64)
	Nose twisted	95.0	3	17	0.15 (0.07, 0.24)	0.64 (0.59, 0.68)
	Mouth	78.5	2	124	0.67 (0.58, 0.74)	0.83 (0.79, 0.85)
	Tongue	92.2	0	71	0.75 (0.71, 0.79)	0.84 (0.81, 0.86)
Total not scored			270	2785		

^†^ Agreement when both observers had scored the behaviour. ^‡^ when observer could not see or score behaviour.

**Table 3:** Univariable associations between Horse Grimace Scale (HGS) scores and lameness (vs. ‘fit to race’), using n = 1500 lateral images of Thoroughbred horses’ heads during in-hand trot-ups of n = 38 racehorses assessed by two independent observers at two time points (40 to 50 days apart).

HGS Parameter		‘Fit to Race’	Lame	Odds Ratio	95% CI	*p*-Value
	Not present	324	200	1.00		
**Stiffly backward ears**	Moderately present	410	362	1.43	0.89, 2.29	0.136
	Obviously present	122	63	0.84	0.23, 3.00	0.784
	Not present	596	417	1.00		
**Orbital tightening**	Moderately present	159	130	1.16	0.56, 2.45	0.680
	Obviously present	44	53	1.72	0.68, 4.34	0.250
	Not present	240	243	1.00		
**Tension above the eye**	Moderately present	402	280	0.69	0.36, 1.33	0.264
	Obviously present	149	67	0.44	0.16, 1.27	0.130
	Not present	344	294	1.00		
**Prominent chewing muscles ^†^**	Moderately present	243	141	0.68	0.35, 1.30	0.242
	Obviously present	39	17	0.51	0.18, 1.41	0.195
	Not present	266	239	1.00		
**Strained nostrils ^‡^**	Moderately present	419	300	0.80	0.47, 1.35	0.399
	Obviously present	82	51	0.69	0.29, 1.65	0.405
	Not present	270	552	1.00		
**Mouth strained ^†^**	Moderately present	339	180	0.51	0.27, 0.97	0.042
	Obviously present	156	89	0.55	0.17, 1.74	0.307
	No pain	405	348	1.00		
	Mild pain	295	177	0.70	0.47, 1.04	0.078
**Overall pain ^§^**	Moderate pain	109	83	0.89	0.46, 1.72	0.720
	Severe pain	38	25	0.77	0.29, 2.04	0.594

^†^ Parameters with >10% missing; ^‡^ parameters with >10% missing and interobserver reliability of <0.4; ^§^ interobserver reliability of <0.4.

**Table 4:** Univariable associations between scores using facial expressions in ridden horses (FEReq) and lameness (vs. ‘fit to race’), using n = 1500 lateral images of Thoroughbred horses’ heads during in-hand trot-ups of n = 38 racehorses assessed by two independent observers at two time points (40 to 50 days apart).

FEReq Parameter		‘Fit to Race’	Lame	Odds Ratio	95% CI	*p*-Value
	Ears forward	257	151	1.00		
	Erect to side	257	226	1.50	0.74, 3.02	0.260
**Ear position**	One ear forward and one ear back	68	46	1.15	0.62, 2.13	0.653
	One ear forward and one erect to side	65	63	1.60	0.88, 2.90	0.121
	One to side and one to back	84	70	1.39	0.59, 3.24	0.453
	Both ears back	117	66	0.94	0.25, 3.54	0.923
**Stare ^§^**	Normal	493	366	1.00		
	Stare	285	207	0.98	0.60, 1.59	0.930
	Not present	593	419	1.00		
**Eye tension**	Moderately present	156	130	1.18	0.56, 2.47	0.661
	Obviously present	42	51	1.72	0.68, 4.35	0.253
**Sclera**	Not exposed	826	582	1.00		
	Exposed	23	54	3.33	1.03, 10.79	0.045
**Eye shape ^‡^**	Round	443	272	1.00		
	Almond	345	320	1.51	0.88, 2.60	0.137
**Tension caudal to eye**	Not present	297	295	1.00		
	Present	501	307	0.62	0.28, 1.37	0.236
**Tension above eye ^§^**	Not present	345	317	1.00		
	Present	435	273	0.68	0.35, 1.33	0.264
	Relaxed	248	229	1.00		
**Nostril tension ^‡^**	Open wide	410	294	0.78	0.45, 1.34	0.364
	Tense	103	61	0.64	0.29, 1.43	0.277
**Wrinkles between nostrils ^‡^**	Not present	401	316	1.00		
	Present	135	76	0.71	0.36, 1.41	0.330
**Wrinkles ventral to nostrils ^§^**	Not present	664	534	1.00		
	Present	139	63	0.56	0.27, 1.19	0.133
	Relaxed	424	395	1.00		
**Upper lip ^§^**	Tense	251	137	0.59	0.30, 1.16	0.126
	Tense and extended	112	42	0.40	0.18, 0.90	0.027
**Lower lip ^†^**	Relaxed	309	300	1.00		
	Tense	428	235	0.57	0.26, 1.23	0.151
**Nose twisted ^§^**	Not present	819	614	1.00		
	Present	28	19	0.91	030, 2.75	0.861
	Closed	567	436	1.00		
	Lips slightly separated: no teeth	113	72	0.83	0.34, 2.03	0.680
	Lips separated, teeth: no gum	35	15	0.56	0.11, 2.93	0.490
	Lips separated, teeth and gum exposed	8	4	0.65	0.08, 5.32	0.688
**Mouth**	Teeth slightly separated: tongue not visible	9	12	1.41	0.35, 8.49	0.497
	Teeth slightly separated: tongue visible	41	16	0.31	0.15, 1.67	0.265
	Mouth widely separated but cannot see tongue	5	4	1.15	0.12, 9.10	0.971
	Teeth widely separated: tongue visible	16	9	0.73	0.11, 4.87	0.747
	Tongue in, not seen	735	553	1.00		
	Tongue seen inside oral cavity	74	32	0.57	0.21, 1.55	0.274
	Tip of tongue protruding, no teeth	13	11	1.12	0.12, 10.80	0.919
**Tongue**	Tip of tongue protruding, can see teeth	4	6	1.99	0.18, 22.52	0.577
	Large part of tongue out, but teeth not exposed	0	0	-	-	-
	Large part of tongue out and teeth exposed	0	1	-	-	-

^†^ Parameters with >10% missing; ^‡^ parameters with >10% missing and interobserver reliability of <0.4; ^§^ interobserver reliability of <0.4.

## Data Availability

The data that support the findings of this study, excluding video-recordings or images that are identifiable, are available from the corresponding author upon reasonable request.

## Supplementary Materials

The following supporting information can be downloaded at: https://www.mdpi.com/article/10.3390/ani13111727/s1, Item S1: Ethogram training manual; References [26,27,30,40] are cited in the supplementary materials; Table S1: Association between the HGS and FEReq and Lameness Locator on a continuous scale, using n = 1500 lateral images of Thoroughbred horses’ heads during in-hand trot-ups of n = 38 racehorses assessed by two independent observers at two time points (40 to 50 days apart).

## Appendix A. Pain Scores for Each Facial Expression Parameter Derived from the Horse Grimace Scale (HGS) and Ethogram Using Facial Expression in Ridden Horses (FEReq)

**Table A1:** Pain scores for each facial expression parameter derived from the Horse Grimace Scale (HGS) and ethogram using facial expression in ridden horses (FEReq) [26,27,30,40].

	Descriptor	Definition	Pain Score
**Head position**	Twisted—nose to left or right	Head tipped to one side of the vertical axis	1
**Ears**	Ears forwards	Both ears forward with pinnae facing forwards	0
	Ears erect and parallel	Both ears vertical with pinnae facing forwards	0
	Both ears erect & to side	Both ears erect and pinnae point to side (divergent), i.e., 180° different directions to the side	2
	One ear forward & one to side	Both ears erect one to the front, and one to the side with pinna pointing to side, 90° different directions	1
	One ear to side & one back	One ear to the side (divergent) and one pinned back—90° different directions	2
	One ear forward & one back	One ear erect and facing forwards and one pinned backwards	1
	Both ears back	Both ears erect pinned back towards the neck	3
**Eyes**	Orbital tightening not present	Eye lids are well apart, not closed	0
	Orbital tightening moderately present	Eye lids are partially closed	1
	Orbital tightening obviously present	Eye lids are almost completely closed	3
	Eye white showing	Sclera exposed	3
	Eye round shape, relaxed	Round-shaped eye	0
	Eye almond shape	Almond/diamond-shaped eye	1
	Tension above the eye	Tension of m. levator anguli oculi medialis	2
	Tension caudal to the eye	Contraction of muscles making zygomatic archmore easily visible	2
	Intense stare	Glazed look	2
**Nostrils**	Relaxed, neutral	Nostril cartilage in neutral position, tear-drop shape	0
	Open wide	Nostril cartilage lifted—mediolateral widening, circular in shape	0
	Tense	Lateral rim of nostril pulled back or down towards the lips, angled sides	1
	Wrinkle between nostrils	Wrinkle between nostrils	1
	Wrinkle between nostril and lip	Wrinkle folds ventral to nostril towards lip	1
**Mouth**	Relaxed and neutral	Lips closed	0
	Lips slightly separated	Lips separated, cannot see teeth	1
	Lips separated some teeth visible	Lips separated can see some teeth, but no gum, and teeth are not apart	1
	Lips separated some teeth visible	Lips separated can see some teeth, but no gum, and teeth are apart	1
	Lips separated and teeth visible but apposed	Lips open showing teeth and gum and teeth which are apposed	2
	Mouth open, teeth exposed and separated, but no tongue	Mouth open, i.e., teeth slightly separated, but cannot see tongue	2
	Mouth open, teeth exposed and widely separated, but tongue not visible	Mouth open, i.e., teeth widely separated, but cannot see tongue	2
	Mouth open, teeth exposed and separated and tongue visible	Mouth open, i.e., teeth widely separated, exposing tongue	3
	Mouth open, teeth exposed and separated and tongue visible	Mouth open, i.e., teeth slightly separated, exposing tongue	2
**Upper lip**	Relaxed	Muzzle relaxed with curved contour in line with lower lip	0
	Muzzle tense	Muzzle tense and angled	1
	Muzzle tense and upper muzzle extended	Muzzle tense and angled and upper muzzle extended	2
**Lower lip**	Relaxed	Muzzle relaxed with curved contour	0
	Muzzle tense	Muzzle tense and angled	1
**Tongue**	Tongue in	Tongue not seen	0
	Tongue seen inside oral cavity	Tongue seen inside oral cavity	1
	Tip of tongue protruding, no teeth	Tip of tongue protruding, cannot see teeth	1
	Tip of tongue protruding, teeth	Tip of tongue protruding, can see teeth	2
	Large part of tongue out, but teeth not exposed	Large part of tongue out, but teeth not exposed	2
	Large part of tongue out and teeth exposed	Large part of tongue out and teeth exposed	3
