# Supplementary material for: Poor Association between Facial Expression and Mild Lameness in Thoroughbred Trot-Up Examinations
Source: Animals (Basel). 2023 May 23;13(11):1727. doi: 10.3390/ani13111727 (PMC10251806; doi:10.3390/ani13111727)
Supplement: Supplementary file 1 [file animals-13-01727-s001.zip › HGS_lameness_Supplementary Table S1.pdf]

Supplementary Table S1. Association between the HGS and FEReq, and Lameness Locator on a continuous scale.

		HDmin			PDmin			HDmax			PDmax		
**Predictor**	**N**	**Coef.**	**95% CI**	**P-value**	**Coef.**	**95% CI**	**P-value**	**Coef.**	**95% CI**	**P-value**	**Coef.**	**95% CI**	**P-value**
**Stiffly backward ears**													
Not present	524	ref			ref			ref			ref		
Moderately present	772	0.71	-1.87,3.30	0.580	0.21	-0.62,1.04	0.607	0.38	-0.93,1.69	0.561	-0.75	-1.57,0.08	0.076
Obviously present	185	-3.40	-7.95,1.15	0.139	1.40	-1.32,4.11	0.304	-0.15	-2.97,2.67	0.917	-1.02	-3.22,1.17	0.352
**Orbital Tightening**													
Not present	1013	ref			ref			ref			ref		
Moderately present	289	-2.36	-6.58,1.86	0.265	0.35	-1.22,1.91	0.655	-1.81	-3.58,-0.47	**0.044**	0.05	-0.84,0.94	0.910
Obviously present	97	-1.45	-5.88,2.98	0.511	-0.28	-2.71,2.15	0.817	-1.66	-4.51,1.19	0.245	-0.33	-1.87,1.21	0.664
**Tension above the eye**													
Not present	483	ref			ref			ref			ref		
Moderately present	682	-1.67	-5.76,2.42	0.413	-0.05	-1.41,1.30	0.936	1.96	0.02,3.90	**0.048**	-0.37	-2.16,1.41	0.675
Obviously present	216	-3.16	-9.46,3.15	0.317	0.15	-1.97,2.27	0.888	2.28	-0.98,5.54	0.165	-0.08	-2.27,2.12	0.945
**Prominent Chewing Muscles****													**0.020**
Not present	638	ref			ref			ref			ref		
Moderately present	384	-4.52	-8.47,-0.56	**0.027**	1.04	-0.09,2.16	0.069	-0.16	-2.03,1.72	0.865	1.28	0.27,2.30	**0.014**
Obviously present	56	-4.42	-8.21,-0.53	**0.027**	0.66	-1.09,2.41	0.447	-0.37	-2.82,2.07	0.758	0.24	-0.94,1.42	0.681
**Strained Nostrils***													

			HDmin			PDmin			HDmax			PDmax	
**Predictor**	**N**	**Coef.**	**95% CI**	**P-value**	**Coef.**	**95% CI**	**P-value**	**Coef.**	**95% CI**	**P-value**	**Coef.**	**95% CI**	**P-value**
Not present	505	ref			ref			ref			ref		
Moderately present	719	-0.17	-3.12,2.78	0.907	-0.16	-1.03,0.72	0.721	0.66	-0.70,2.02	0.331	-0.67	-1.70,0.37	0.201
Obviously present	133	-2.92	-6.92,1.08	0.148	-0.56	-2.07,0.95	0.457	-0.46	-2.68,1.76	0.676	-0.32	-1.94,1.30	0.689
**Mouth Strained****													
Not present	552	ref			ref			ref			ref		
Moderately present	519	-1.74	-6.09,2.62	0.425	-0.66	-2.04,0.71	0.335	0.02	-1.65,1.67	0.989	-1.21	-3.21,0.80	0.231
Obviously present	245	-2.10	-8.36,4.16	0.501	-0.71	-3.02,1.60	0.538	0.92	-2.12,3.95	0.541	-1.46	-3.84,0.92	0.221
**Overall Pain***													
No Pain	753	ref			ref			ref			ref		
Mild Pain	472	-2.13	-4.09,-0.17	**0.034**	-0.15	-0.85,0.54	0.663	0.18	-0.83,1.19	0.723	-0.57	-1.81,0.67	0.356
Moderate Pain	192	-0.93	-4.22,2.36	0.569	-0.12	-1.48,1.23	0.856	1.00	-0.90,2.88	0.293	-0.76	-1.98,0.45	0.212
Severe Pain	63	-3.17	-8.15,1.82	0.206	0.04	-2.11,0.42	0.968	1.01	-1.45,3.49	0.408	-1.24	-3.18,0.71	0.206
**Ear Position**													
Ears Forward	408	ref			ref			ref			ref		
Erect to side	483	1.06	-2.38,4.51	0.537	0.04	-1.05,1.13	0.936	0.60	-1.41,2.62	0.548	-1.16	-2.15	0.024
One ear forward, one ear back	114	1.15	-2.10,4.41	0.476	-0.04	-0.90,0.81	0.919	0.28	-1.43,1.98	0.744	-0.20	-1.16	0.676
One ear forward, one erect to side	130	2.09	-2.08,6.26	0.317	0.73	-0.45,1.91	0.220	0.74	-1.12,2.60	0.425	0.88	-1.33	0.425
One to side, one to back	156	-1.45	-6.60,3.70	0.571	1.00	-0.70,2.70	0.241	0.04	-2.17,2.26	0.966	-1.08	-2.72	0.188

		HDmin			PDmin			HDmax			PDmax		
**Predictor**	**N**	**Coef.**	**95% CI**	**P-value**	**Coef.**	**95% CI**	**P-value**	**Coef.**	**95% CI**	**P-value**	**Coef.**	**95% CI**	**P-value**
Both ears back	186	-3.23	-8.10,1.65	0.188	1.37	-1.23,4.33	0.266	-0.06	-3.05,2.92	0.964	-1.07	-3.21	0.319
**Stare***													
Normal	860	ref			ref			ref			ref		
Stare	492	-0.65	-2.80,1.50	0.545	0.08	-0.76,0.91	0.854	0.78	-0.53,2.09	0.237	-0.26	-1.01,0.49	0.494
**Eye Tension**													
Not present	1012	ref			ref			ref			ref		
Moderately present	286	-2.35	-6.61,1.91	0.271	0.40	-1.21,2.01	0.619	-1.73	-3.50,0.03	**0.054**	0.14	-0.81,1.08	0.771
Obviously present	93	-1.41	-5.87,3.06	0.527	-0.42	-2.81,1.97	0.723	-1.60	-4.47,1.26	0.262	-0.45	-2.00,1.09	0.556
**Sclera**													
Not exposed	1408	ref			ref			ref			ref		
Exposed	77	8.99	-2.69,20.68	0.127	0.21	-0.95,1.37	0.883	2.43	-2.16,7.03	0.289	-1.00	-2.48,-.48	0.179
**Eye Shape***													
Round	716	ref			ref			ref			ref		
Almond	665	-1.00	-3.51,1.51	0.425	0.21	-0.95,1.37	0.715	-1.30	-2.83,0.24	0.096	0.43	-0.52,1.37	0.366
**Tension caudal to eye**													
Not present	592	ref			ref			ref			ref		
Present	808	-3.24	-8.08,1.59	0.182	0.44	-0.92,1.81	0.514	0.74	-1.57,3.04	0.522	0.68	-0.60,1.95	0.291
**Tension above eye***													

		HDmin			PDmin			HDmax			PDmax		
**Predictor**	**N**	**Coef.**	**95% CI**	**P-value**	**Coef.**	**95% CI**	**P-value**	**Coef.**	**95% CI**	**P-value**	**Coef.**	**95% CI**	**P-value**
Not present	662	ref			ref			ref			ref		
Present	708	-0.55	-4.6,3.47	0.782	-0.38	-1.61,0.85	0.536	2.19	0.18,4.20	**0.034**	-0.46	-2.04,1.12	0.559
**Strained Nostrils*** **													
Relaxed	478	ref			ref			ref			ref		
Circular	705	-0.35	-3.56,2.85	0.824	-0.10	-1.05,0.84	0.827	0.69	-0.60,1.99	0.286	-0.79	-2.11,0.53	0.234
Tense	164	-3.36	-7.36,0.63	0.096	-0.40	-1.84,1.05	0.582	-0.64	-2.82,1.54	0.553	-0.30	-1.77,1.18	0.686
**Wrinkles between nostrils*** **													
Not present	717	ref			ref			ref			ref		
Present	211	-2.80	-8.00,2.40	0.282	-0.49	-1.35,0.37	0.251	0.26	-1.60,2.12	0.775	-0.20	-0.93,0.54	0.593
**Wrinkles Ventral to Nostrils* **													
Not present	1198	ref			ref			ref			ref		
Present	202	-3.77	-7.23,-0.31	**0.034**	0.06	-1.20,1.32	0.924	-0.46	-2.54,1.61	0.652	0.25	-1.20,1.71	0.729
**Upper Lip* **													
Relaxed	821	ref			ref			ref			ref		
Tense	391	-2.89	-6.73,0.96	0.137	-0.34	-1.57,1.10	0.726	-0.59	-2.32,1.14	0.495	-0.53	-1.76,0.70	0.386
Tense and extended	154	-1.10	-4.22,2.03	0.508	-0.94	-2.46,0.58	0.220	-0.16	-2.39,2.06	0.882	-0.55	-1.77,0.67	0.364
**Lower Lip*** **													
Relaxed	611	ref			ref			ref			ref		

		HDmin			PDmin			HDmax			PDmax		
**Predictor**	**N**	**Coef.**	**95% CI**	**P-value**	**Coef.**	**95% CI**	**P-value**	**Coef.**	**95% CI**	**P-value**	**Coef.**	**95% CI**	**P-value**
Tense	663	-1.96	-7.03,3.11	0.438	-0.44	-1.93,1.04	0.549	0.13	-1.90,2.16	0.898	-1.12	-3.06,0.81	0.247
**Nose Twisted***													
Not twisted	1443	ref			ref			ref			ref		
Twisted	47	8.39	-1.97,18.75	0.109	-2.51	-3.84,-1.19	**0.000**	3.57	0.05,7.08	**0.047**	-0.87	-2.58,0.85	0.312
**Mouth**													
Closed	1006	ref			ref			ref			ref		
Lips slightly separated: no teeth	185	-2.64	-7.45,2.18	0.275	-0.37	-1.94,1.19	0.631	0.24	-1.90,2.37	0.824	-1.30	-2.77,0.17	0.082
Lips separated, teeth: no gum	52	-2.86	-10.26,4.54	0.493	0.23	-2.83,3.29	0.878	1.47	-1.90,4.84	0.381	-1.80	-3.72,-0.12	**0.065**
Lips separated, teeth and gum exposed	13	-2.45	-7.84,2.95	0.407	0.12	-4.43,4.67	0.957	5.51	1.55,9.47	**0.008**	-3.07	-5.36,-0.77	**0.010**
Teeth slightly separated: tongue not visible	23	0.43	-6.11,7.98	0.777	1.17	-3.04,5.37	0.578	3.11	-0.27,6.48	**0.070**	-1.25	-3.03,0.52	0.161
Teeth slightly separated: tongue visible	58	-2.94	-8.29,2.41	0.286	-0.28	-1.98,1.42	0.738	0.80	-1.92,3.51	0.556	-1.25	-3.17,0.71	0.206
Mouth widely separated but cannot see tongue	10	-3.03	-9.97,3.91	0.593	0.35	-4.59,5.29	0.886	4.46	-0.02,8.93	**0.051**	-2.46	-4.65,-0.26	**0.029**
Teeth widely separated: tongue visible	27	-3.74	-8.83,1.35	0.108	-0.59	-3.60,2.43	0.695	3.20	-2.43,8.83	0.256	-1.77	-5.03,1.49	0.278
**Tongue**													
Tongue in, not visible	1288	ref			ref			ref			ref		
Tongue seen inside oral cavity	106	-3.11	-7.43,1.21	0.153	-0.38	-1.90,1.14	0.616	0.26	-2.52,3.04	0.849	-1.33	-3.04,0.39	0.125
Tip of tongue protruding, no teeth	24	-6.19	-10.76,-1.62	**0.009**	0.85	-3.03,4.73	0.661	-1.09	-4.56,2.38	0.529	-0.22	-4.17,3.72	0.910

		HDmin			PDmin			HDmax			PDmax		
**Predictor**	**N**	**Coef.**	**95% CI**	**P-value**	**Coef.**	**95% CI**	**P-value**	**Coef.**	**95% CI**	**P-value**	**Coef.**	**95% CI**	**P-value**
Tip of tongue protruding, can see teeth	10	-8.63	-13.02,-4.25	**0.000**	1.72	-1.76,5.19	0.324	-0.75	-6.38,4.88	0.789	1.09	-2.09,4.27	0.491
Large part of tongue out, but teeth not exposed	0												
Large part of tongue out and teeth exposed	1	-8.87	-12.75,-5.00	**0.000**	3.90	3.17,5.63	**0.000**	-4.11	-5.93,-2.29	**0.000**	2.73	1.39,4.07	**0.000**

*interobserver reliability of <0.40; ** parameters with >10% missing; ***parameters with >10% missing and inter-reliability of <0.40
