# Supplementary material for: Poor Association between Facial Expression and Mild Lameness in Thoroughbred Trot-Up Examinations
Source: Animals (Basel). 2023 May 23;13(11):1727. doi: 10.3390/ani13111727 (PMC10251806; doi:10.3390/ani13111727)
Supplement: Supplementary file 1 [file animals-13-01727-s001.zip › Supplementary Item S1. Ethogram training manual.pdf]

Ethogram Training Method:

not present (0); moderately present (1); obviously present (2).

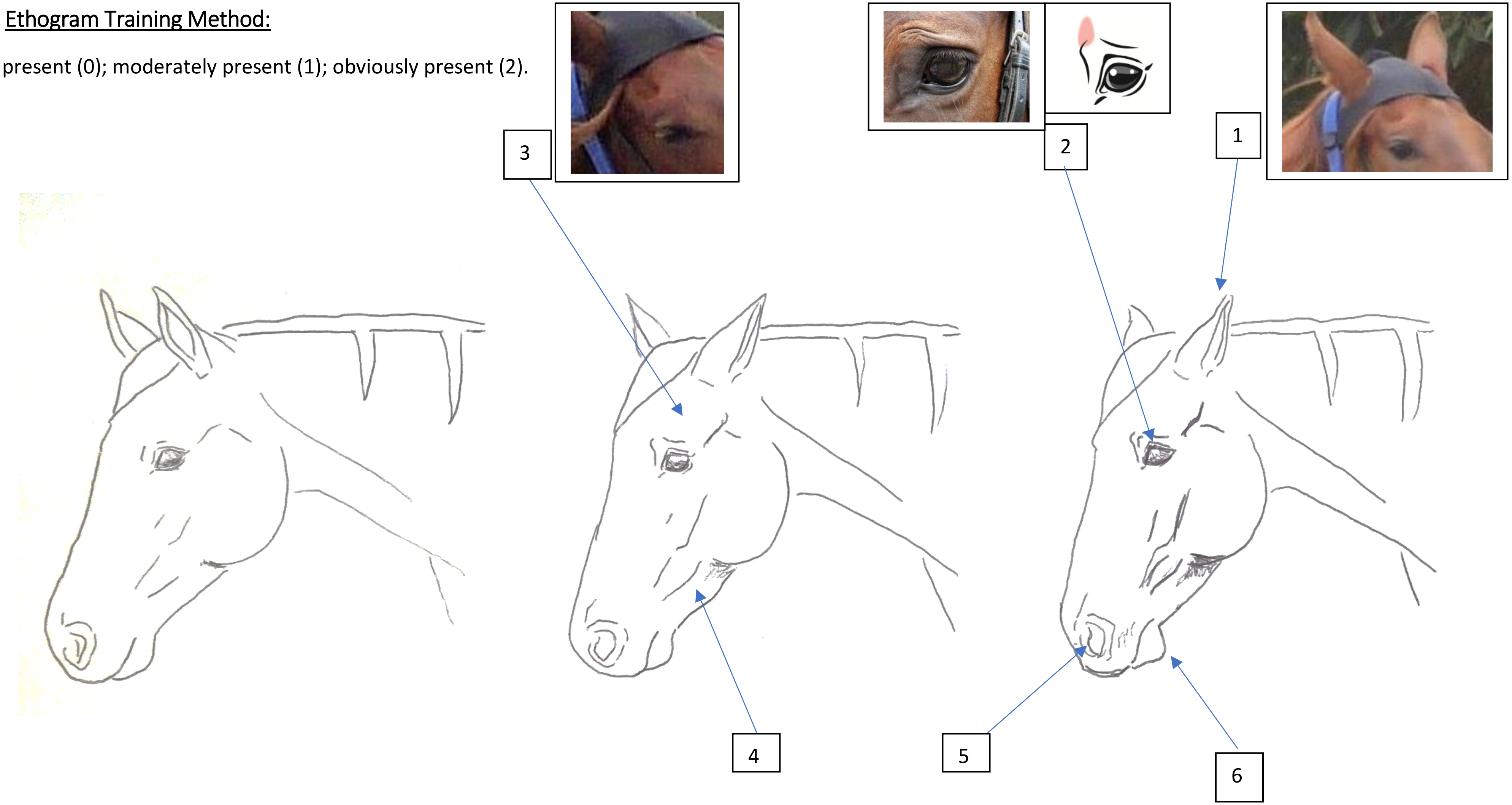

Modified pictorial ethogram described by (Gleerup, Forkman, Lindegaard, & Andersen, 2015), supporting information for Horse Grimace Scale (HGS) (Dalla Costa et al., 2014)

1. Stiffly backward ears – *space between ears wider relative to baseline*
2. Orbital tightening – *eyelid is partially or completely closed; eye reduced more than half is obviously present.*
3. Tension above the eye – *contraction of the muscles above eye; increased visibility of underlying bone surfaces.*
4. Prominent chewing muscles – *straining chewing muscles clearly visible as an increase tension above mouth.*
5. Strained nostrils – *nostrils look strained and slightly dilated, profile of nose flattens, and lips elongate.*
6. Mouth strained and pronounced chin – *strained mouth is clearly visible when upper lip is drawn back, pronounced chin.*

Variables described by (Dyson, Berger, Ellis, & Mullard, 2017; Gleerup et al., 2015; Mullard, Berger, Ellis, & Dyson, 2017)

Ear position:

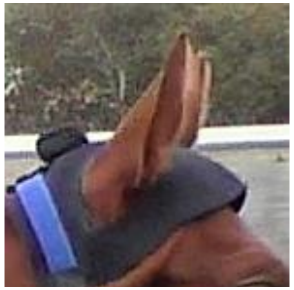

Ears Forward –

Both ears forward with pinnae facing forward

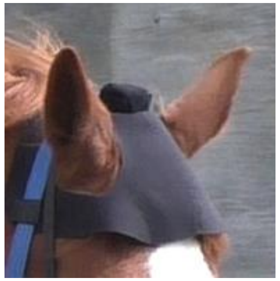

Erect to side –

Both ears erect, and pinnae point to side (divergent) i.e. 180° different directions to the side

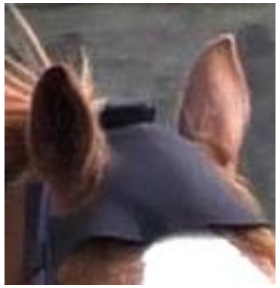

One ear forward and one ear back -

One ear pinnae facing forward and one back towards the neck

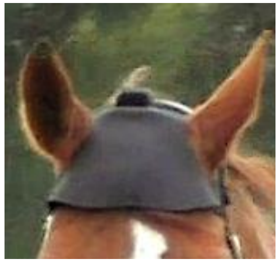

One forward and one erect & to side –

both ears erect one to the front, and one to the side with pinna pointing to side, 90° different directions

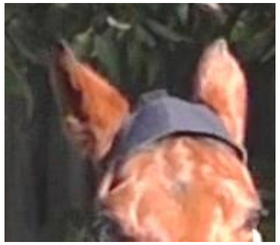

One to side and one to back –

One ear to the side (divergent) and one pinned back - 90° different directions

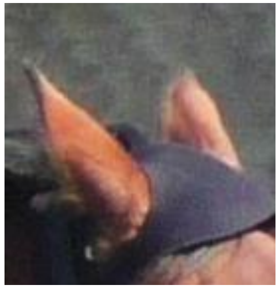

Both ears back –

Both ears erect pinned back towards the neck

Expression:

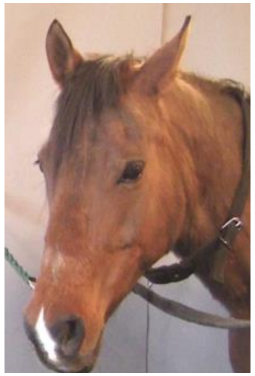

Relaxed

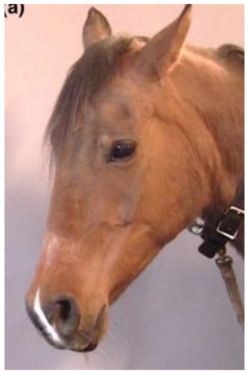

Intense/glazed look

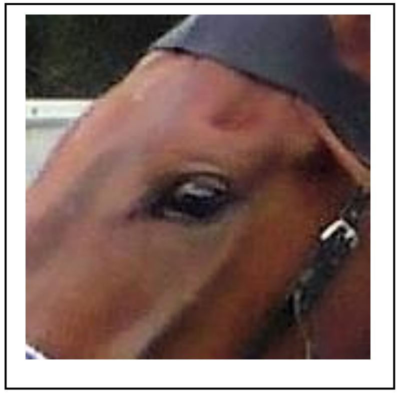

Sclera exposed –

White surround eye is visible caused by tension of the levator palpebrae superioris muscle (which elevates the upper eyelid)

Eye shape:

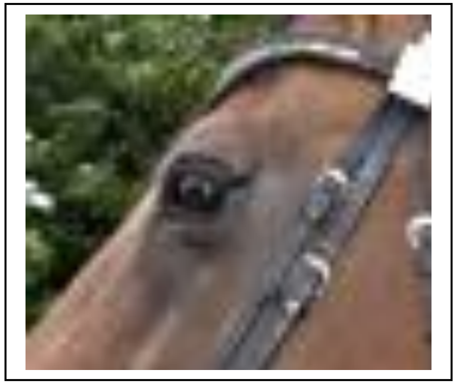

Round Eye

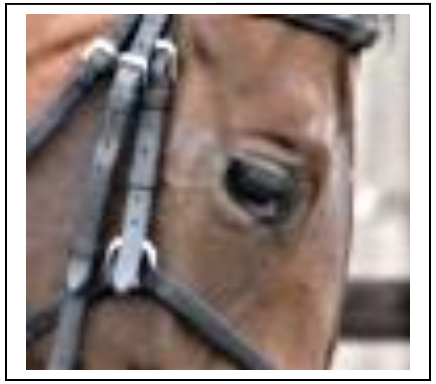

Almond Eye

Orbital tightening:

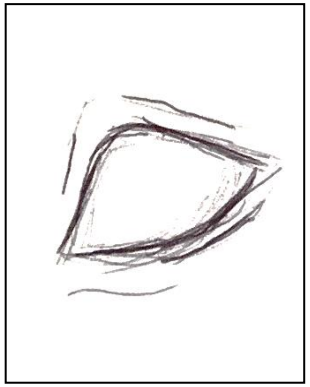

Not present

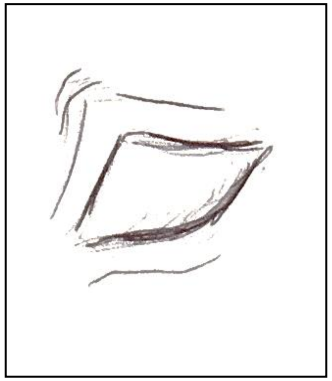

Moderately present –

Eye is partially closed

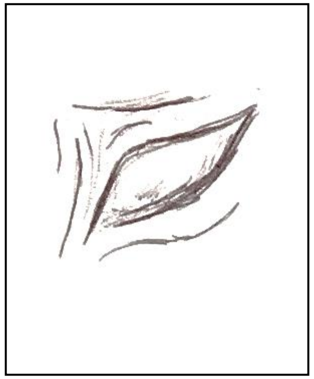

Obviously present –

Eye reduced more than half

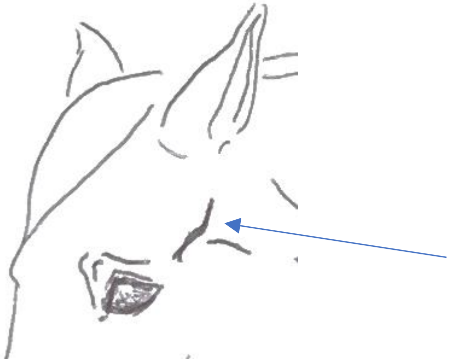

Tension caudal to the eye - *The zygomatic arch is more obvious because of tension in the muscles caudal to the eye.*

Nostrils:

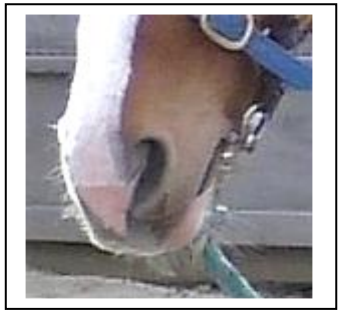

Relaxed/tear drop

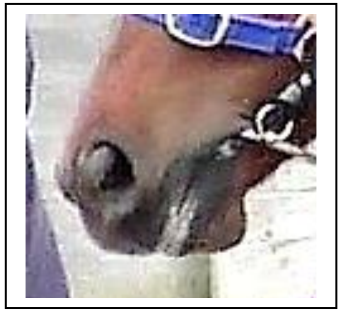

Open wide, circular shape

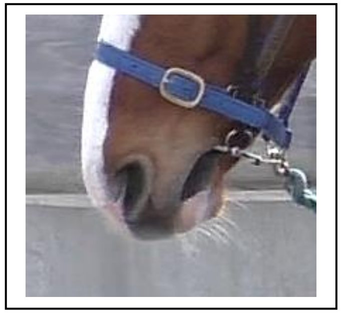

Tense

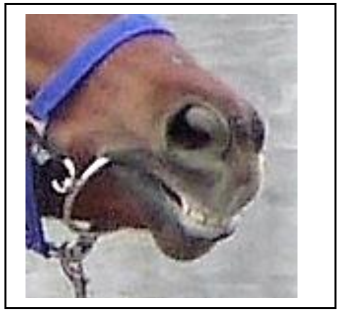

Wrinkle between nostrils present

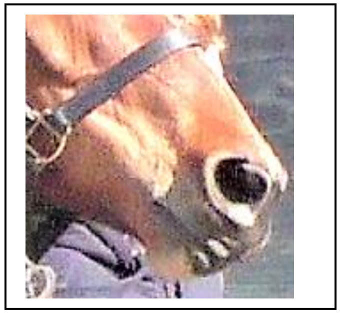

Wrinkle between folds ventral to nostril towards lip present

Upper Lip:

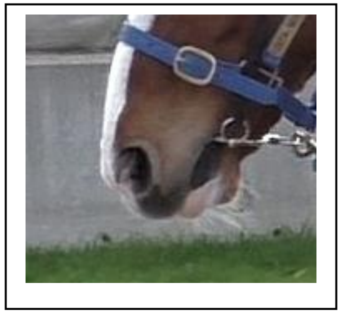

Relaxed –

Muzzle relaxed with curved contour in line with lower lip

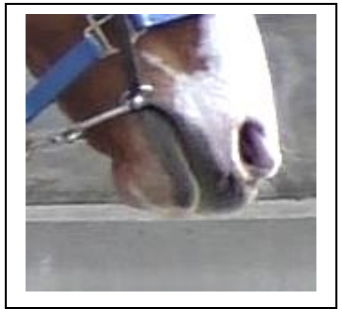

Tense –

Muzzle tense and angled

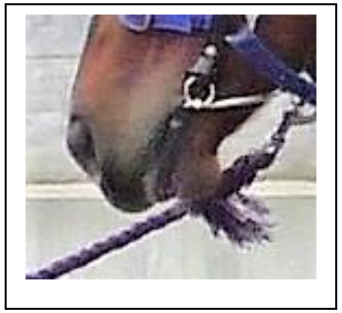

Tense and extended–

Muzzle tense and angled and upper muzzle extended

Lower Lip:

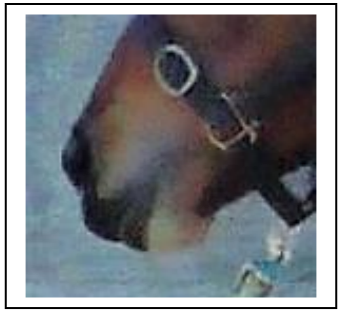

Relaxed –

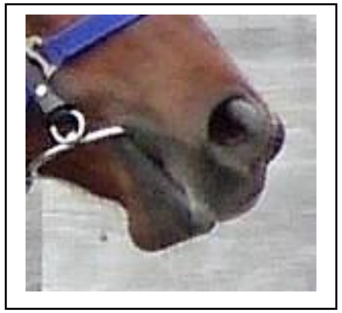

Tense –

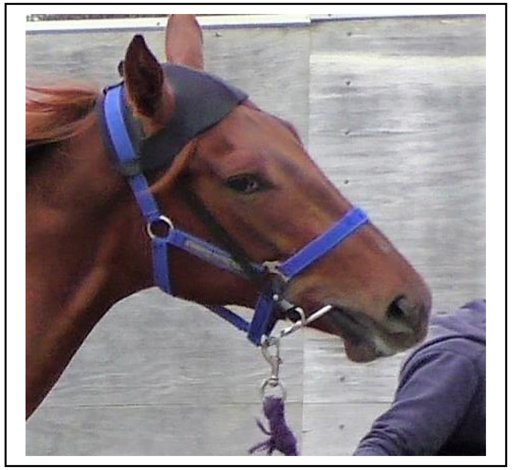

Direction of head –

Twisted nose to one side

References:

Dalla Costa, E., Minero, M., Lebelt, D., Stucke, D., Canali, E., & Leach, M. C. (2014). Development of the Horse Grimace Scale (HGS) as a Pain Assessment Tool in Horses Undergoing Routine Castration. *PLOS ONE, 9*(3), e92281. doi:10.1371/journal.pone.0092281

Dyson, S., Berger, J. M., Ellis, A. D., & Mullard, J. (2017). Can the presence of musculoskeletal pain be determined from the facial expressions of ridden horses (FEReq)? *Journal of Veterinary Behavior: Clinical Applications and Research, 19*, 78-89. doi:https://doi.org/10.1016/j.jveb.2017.03.005

Gleerup, K. B., Forkman, B., Lindegaard, C., & Andersen, P. H. (2015). An equine pain face. *Veterinary Anaesthesia and Analgesia, 42*(1), 103-114. doi:10.1111/vaa.12212

Mullard, J., Berger, J. M., Ellis, A. D., & Dyson, S. (2017). Development of an ethogram to describe facial expressions in ridden horses (FEReq). *Journal of Veterinary Behavior, 18*, 7-12. doi:https://doi.org/10.1016/j.jveb.2016.11.005
